# Ack promotes tissue growth via phosphorylation and suppression of the Hippo pathway component Expanded

**DOI:** 10.1038/celldisc.2015.47

**Published:** 2016-02-23

**Authors:** Lianxin Hu, Jiajun Xu, Meng-Xin Yin, Liguo Zhang, Yi Lu, Wenqing Wu, Zhaoyu Xue, Margaret S Ho, Guanjun Gao, Yun Zhao, Lei Zhang

**Affiliations:** 1 State Key Laboratory of Cell Biology, Innovation Center for Cell Signaling Network, Institute of Biochemistry and Cell Biology, Shanghai Institutes for Biological Sciences, Chinese Academy of Sciences, Shanghai, China; 2 Department of Cell and Developmental Biology, University of Illinois at Urbana-Champaign, Urbana, IL, USA; 3 School of Life Sciences, Tsinghua University, Beijing, China; 4 Department of Anatomy and Neurobiology, School of Medicine, Tongji University, Shanghai, China; 5 School of Life Science and Technology, ShanghaiTech University, Shanghai, China

**Keywords:** Hippo pathway, activated cdc43 kinase, Yorkie, expanded

## Abstract

Non-receptor tyrosine kinase activated cdc42 kinase was reported to participate in several types of cancers in mammals. It is also believed to have an anti-apoptotic function in *Drosophila*. Here, we report the identification of *Drosophila* activated cdc42 kinase as a growth promoter and a novel Hippo signaling pathway regulator. We find that activated cdc42 kinase promotes tissue growth through modulating Yorkie activity. Furthermore, we demonstrate that activated cdc42 kinase interacts with Expanded and induces tyrosine phosphorylation of Expanded on multiple sites. We propose a model that activated cdc42 kinase negatively regulates Expanded by changing its phosphorylation status to promote tissue growth. Moreover, we show that *ack* genetically interacts with *merlin* and *expanded*. Thus, we identify *Drosophila* activated cdc42 kinase as a Hippo pathway regulator.

## Introduction

**A**ctivated **c**dc42 **k**inase (Ack) belongs to a conserved family of non-receptor tyrosine kinase [1]. Several studies suggested that human ACK1 has important roles during the progression of several cancers. It is known that ACK1 activation is associated with poor prognosis and metastatic phenotypes in human tumors [2]. Advances in genome sequencing have revealed several ACK1 somatic mutations; however, gene amplification is a more important mechanism of ACK1 activation, which has been observed in many types of cancers (COSMIC and cBioPortal databases) [3]. During prostate cancer progression, ACK1 can phosphorylate tumor suppressor Wwox to promote Wwox’s degradation. It also phosphorylates androgen receptor that modulates the radiation resistance of cancer cells [4–6]. Despite above findings, Ack might function in a more complicated way rather than a simple participation in the development of spontaneous tumors [7]. In the genetically tractable animal organism *Drosophila*, Cdc42 signaling that leads to dorsal closure of the epidermis during embryogenesis has been shown to regulate Ack protein level [8]. Moreover, Ack has been reported to regulate Dock localization in male germ cells during spermatogenesis [9]. Notwithstanding these findings, the function of *Drosophila* Ack during tumorigenesis remains elusive. Not until recently, a study reported that Ack has an anti-apoptotic function, and it genetically interacts with Yorkie (Yki) to regulate cell proliferation and apoptosis [10].

The Hippo pathway controls organ size by orchestrating proliferation and apoptosis in diverse cells and tissues [11–13]. Most, if not all, of the Hippo pathway’s functions are exhibited through its downstream transcriptional coactivator Yki [14]. The activity of Yki is closely related with its localization. The localization of Yki can be fine-tuned by the Hippo pathway either through modulation of its phosphorylation status or through physical interactions [15, 16]. Central to the Hippo signaling pathway is a kinase cascade, the best demonstration of phosphorylation-dependent Yki regulation. The STE20 family serine/threonine kinase Hippo (Hpo) [17–21], activated by autophosphorylation and dimerization [22, 23], phosphorylates and activates the NDR family kinase Warts (Wts) [22, 24, 25], leading to Wts-mediated Yki phosphorylation. The adaptor proteins Salvador (Sav) [26, 27] and Mob as tumor suppressor (Mats) [28] facilitate the activities of Hpo and Wts. Phosphorylated Yki is captured by 14-3-3 protein and retained in cytosol; thus, its activity is inhibited [24, 25, 29, 30]. Expanded (Ex) is an important upstream component in the Hippo pathway. Ex, together with Merlin (Mer) and Kibra, transduces signals to the Hpo-Wts kinase cascade through multiple protein interactions [31]. In addition, Ex interacts with Yki through WW domain and PPxY motif to restrict Yki in cytosol [32, 33]. WW domain and PPXY/PY motif interactions participate in the regulation of Yki [34, 35]. For instance, WBP2 interacts with Yki’s WW domains through its PY motifs to promote Yki’s activity [36, 37].

In this study, we report the identification of *Drosophila* Ack as a growth promoter and a novel Hippo signaling pathway regulator. We show that Ack promotes tissue growth through enhancing Yki activity. Furthermore, we demonstrate that Ack interacts with Ex and negatively regulates Ex activity in a kinase-dependent manner by changing its phosphorylation status on multiple tyrosine residues. In addition, we determine that *ack* genetically interacts with *merlin* and *ex*. Taken together, our findings support that *Drosophila* Ack is a Hippo pathway regulator and promotes tissue growth via suppressing Ex-mediated Yki regulation.

## Results

### Ack overexpression upregulates Yki activity to promote growth

**Figure 1:**
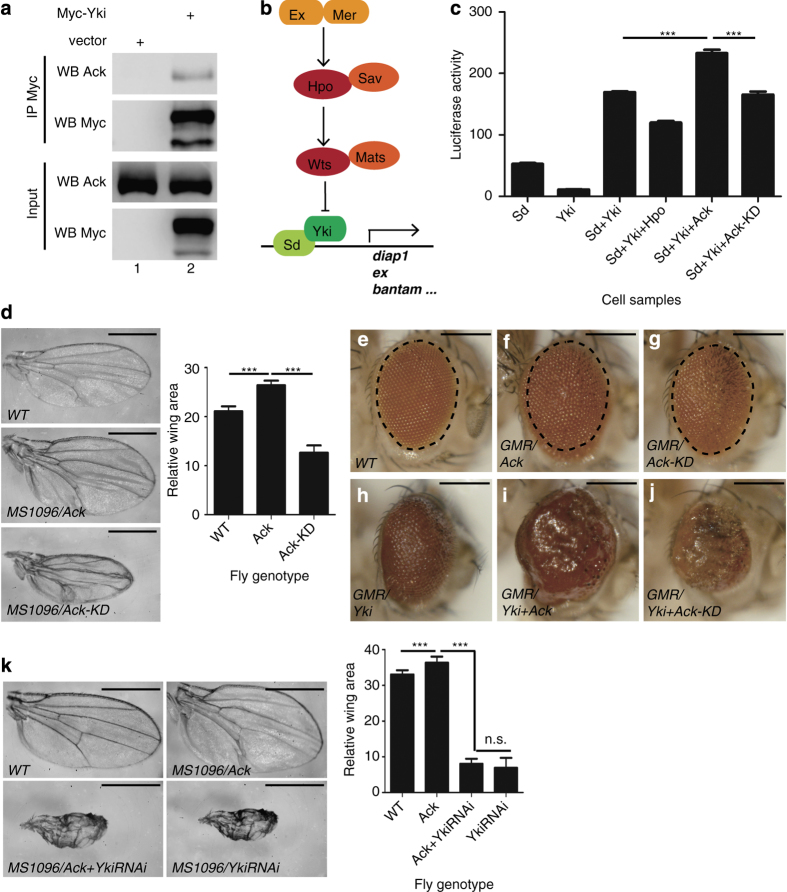
Ack interacts with Yki and promotes tissue growth. (**a**) Ack interacts with Yki. S2 cells expressing Myc-Yki or blank vector were immunoprecipitated and probed with indicated antibodies. Experiment was repeated three times; and representative blots are shown. (**b**) A schematic of Hippo signaling pathway. (**c**) Ack promotes the transcriptional activity of the Yki–Sd complex in a kinase-dependent manner. S2 cells were transfected with the indicated constructs; and the cell lysates were subjected to dual luciferase assay. Quantitative data are expressed as mean±s.d. (triplicate wells). ***P<0.001. (**d**) Ack overexpression promotes tissue growth *in vivo*. Wild-type male wing and wings expressing *MS1096-Gal4* with *UAS-V5-Ack* or *UAS-V5-Ack-KD* were shown. Ack and Ack-KD were inserted at the same *attp* site to ensure equal expression level. Overexpression of Ack induces wing overgrowth compared with control, whereas overexpression of Ack-KD suppresses wing growth. Quantification of wing area of each group is expressed as mean±s.d. (*N*=10). ***P<0.001. Scale bar is 500 μm. (**e**–**j**) Ack promotes eye growth and enhances Yki activity. Wild-type adult eye (**e**) or eyes expressing the indicated constructs (**f**–**j**) were shown. Dashed line indicated normal eye size. Scale bar is 300 μm. Experiments were repeated; and representative eyes are shown. (**k**) Yki RNAi suppresses Ack-induced wing growth. Wild-type male wings and wings expressing indicated constructs were shown. Quantification of wing area of each group is expressed as mean±s.d. (*N*=10). ***P<0.001. n.s., not significant. Scale bar is 500 μm.

To identify novel regulators of the Hippo pathway, we performed mass spectrometry (MS) analysis using GST fused Yki tandem WW domains (refer to as GST-WW) as bait to identify its binding partners. As expected, some known Yki binding partners such as Wts, WWBP, Mop and Ack were found in the result (Supplementary Figure S1). An interaction of Ack and Yki was reported recently [10, 38], which was confirmed using co-immunoprecipitation (co-IP) experiment (Figure 1a).

Yki is the downstream Hippo pathway effector, and its activity is tightly regulated by upstream Hippo pathway components via inducing Yki Ser168 phosphorylation or physical interaction (Figure 1b) [39]. We then sought to determine whether Ack affects Yki activity. A dual luciferase assay was established to monitor the transcriptional activity of Scalloped (Sd)–Yki complex [40]. Coexpression of Yki and Sd in S2 cells activated the reporter gene (*3×Sd2-Luc*), which was promoted by Ack expression (Figure 1c). Coexpression of a kinase-dead form of Ack (Ack-KD, which contains a lysine to arginine substitution at amino acid 156) [8] could not promote Sd–Yki complex activity as wild-type Ack (Figure 1c), indicating that the kinase activity of Ack is essential for its function on promoting Sd–Yki transcriptional activity *in vitro*.

To verify whether Ack promotes Yki activity *in vivo*, we generated Ack transgenic flies using *Gal4/UAS* system [41]. Overexpression of Ack (but not Ack-KD) under the control of *MS1096-Gal4* or *GMR-Gal4* promoted growth in wings and eyes, respectively (Figure 1d–g). In addition, coexpression of Ack (but not Ack-KD) further promoted Yki-induced eye overgrowth (Figure 1h–j). Knockdown of Yki by RNAi suppressed Ack overexpression-induced overgrowth phenotype, generating phenotypes similar to that induced by depletion of Yki (Figure 1k). On the basis of these results, we speculated that Ack promoted tissue overgrowth through upregulating Yki’s activity.

### Ack overexpression upregulates the Hippo pathway targets

**Figure 2:**
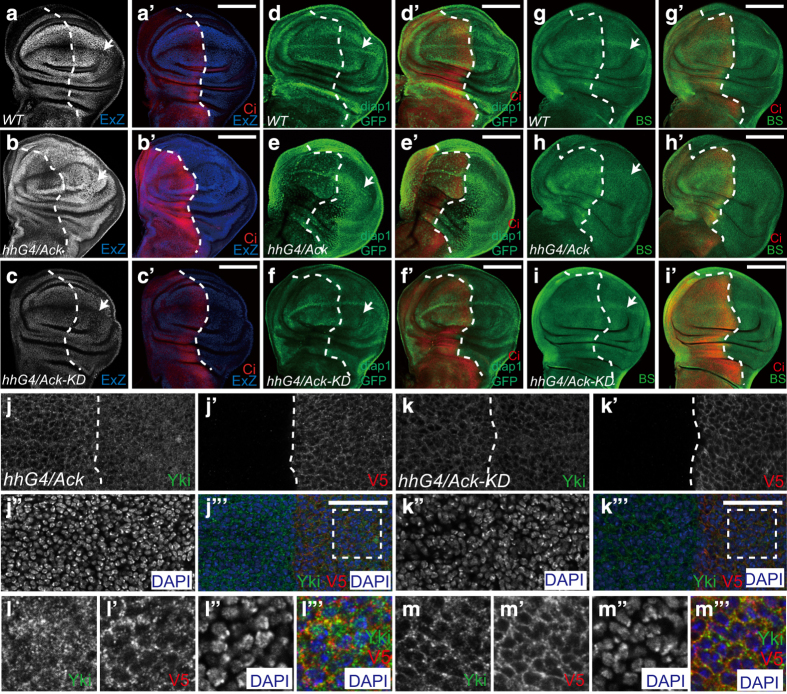
Ack overexpression upregulates Hippo pathway targets. (**a**–**i′**) Ack overexpression upregulates Yki target genes. Wild-type wing discs (**a**, **a′**, **d**, **d′** and **g**, **g′**) or wing discs expressing *UAS-V5-Ack* (**b**, **b′**, **e**, **e′** and **h**, **h′**) or *UAS-V5-Ack-KD* (**c**, **c′**, **f**, **f′** and **i**, **i′**) by *hh-Gal4* were immunostained to show the expression of Ci and Hippo pathway markers, including *Ex-lacZ* (**a**–**c′**), *diap1-GFP4.3* (**d**–**f′**) and *bantam* sensor (BS) GFP. Note that, in the P compartment, Ack overexpression upregulates the expression of Ex-lacZ and diap1-GFP and downregulates BS GFP, while Ack-KD overexpression cannot (**g**–**i′**). Arrows indicated the P compartment. White dashed lines indicated the A-P compartment boundary based on Ci staining. Scale bar is 100 μm. (**j**–**k′′′**) *Drosophila* wing discs expressing *UAS-V5-Ack* (**j**–**j′′′**) or *UAS-V5-Ack-KD* (**k**–**k′′′**) by *hh-Gal4* were immunostained with Yki and V5 antibody. Compared with the A compartment, in the P compartment, Ack overexpression promotes Yki nuclear localization whereas Ack-KD overexpression cannot. **l**–**l′′′** and **m**–**m′′′** are magnified pictures of the dashed line indicated P compartment area in **j**–**j′′′** and **k**–**k′′′**, respectively. DAPI was stained to show nuclei. White dashed line indicated the A-P compartment boundary. Scale bar is 20 μm.

To confirm our speculation, Ack expression was driven in posterior (P) compartment of wing discs using *hedgehog-Gal4* (*hh-Gal4*); and the expression level of Yki target genes was evaluated. *ex* is one of the Yki target genes and encodes an upstream Hippo pathway component that suppresses Yki’s function [42]. Its transcription level can be reflected by *Ex-LacZ* (*Ex-Z*). Another well-known Yki target gene is *diap1*, whose transcriptional level can be monitored using a reporter gene *diap1-GFP4.3 *[40]. Our results showed that overexpression of Ack using *hh-Gal4* enhanced *Ex-Z* signal and induced a significant upregulation of *diap1-GFP* signal in the P compartment (Figure 2a–b′ and d–e′). Furthermore, microRNA *bantam*, a previously reported Yki target, has been shown to control cell proliferation and apoptosis [43, 44]. As expected, the expression of its reporter, Bantam sensor GFP, that reversely correlates with the microRNA *bantam* expression, was downregulated in the P compartment when Ack was overexpressed using *hh-Gal4* (Figure 2g–h′), indicating an elevation of *bantam* transcription. In addition, the P compartment of wing discs was enlarged in size when Yki target gene expression was elevated by Ack overexpression (Figure 2b–b′, e–e′ and h–h′). In contrast, overexpression of Ack-KD could not promote Yki target gene expression (Figure 2c–c′, f–f′ and i–i′). Together, these findings indicate that the regulation of Yki activity by Ack is kinase dependent *in vivo*.

Notably, cytoplasmic-nuclear translocation of endogenous Yki was affected when Ack (but not Ack-KD) was overexpressed (Figure 2j–k′′′). Yki was increasingly accumulated in the nucleus upon Ack overexpression (but not Ack-KD expression) (Figure 2l–m′′′), and this increase is correlated with the upregulation of its activity. Collectively, these results indicate that Ack overexpression enhances Yki activity in a kinase activity-dependent manner *in vitro* and *in vivo*.

### Ack modulates Yki activity via regulating Ex activity

**Figure 3:**
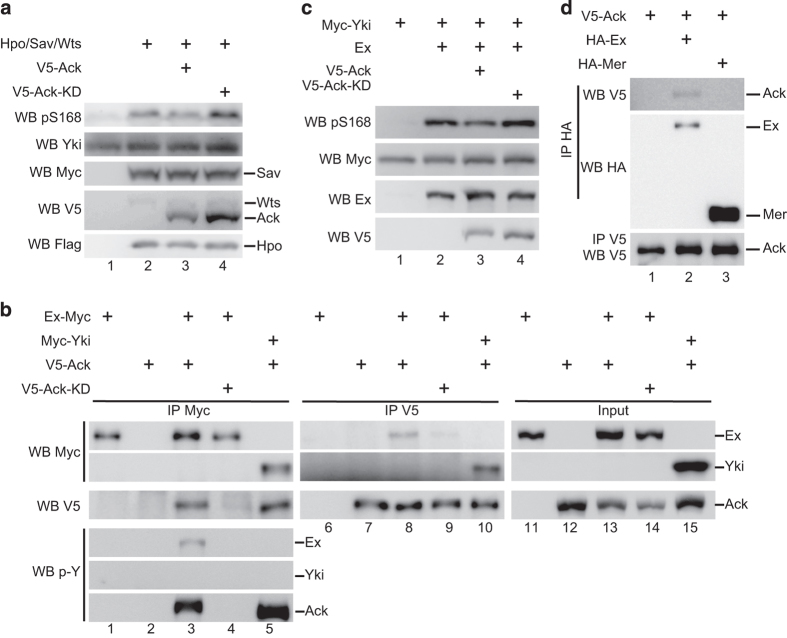
Ack interacts with and phosphorylates Ex. (**a**) Ack overexpression suppresses the upregulation of Yki S168 phosphorylation induced by Hpo/Sav/Wts. S2 cells expressing the indicated constructs were probed with the indicated antibody. Experiment was repeated three times, and representative blots are shown. (**b**) Ex interacts with Ack, and is phosphorylated by Ack. S2 cells expressing the indicated constructs were immunoprecipitated and probed with the indicated antibodies. Experiment was repeated three times, and representative blots are shown. (**c**) Ack inhibits Ex-induced Yki S168 phosphorylation. S2 cells expressing indicated constructs were probed with the indicated antibody. Note that Ex-induced upregulation of pS168 is inhibited by Ack but not by Ack-KD. Experiment was repeated three times, and representative blots are shown. (**d**) Ex interacts with Ack, while Mer does not interact with Ack. S2 cells expressing the indicated constructs were immunoprecipitated and probed with the indicated antibodies. Experiment was repeated three times, and representative blots are shown.

To shed some light on possible mechanisms by which Ack promotes growth, we performed western blot and co-IP analyses. As we noticed that Ack interacted with Yki in S2 cells and overexpression of Ack induced Yki nuclear translocation *in vivo* (Figures 1a and 2j–j′′′), we speculated that Ack promotes Yki’s activity directly. However, in S2 cells, Ack overexpression could not induce tyrosine phosphorylation of Yki (described later in Figure 3b). Ack expression slightly suppressed Hippo signaling-induced upregulation of Yki Ser168 phosphorylation (pS168) (Figure 3a), implying that Ack-induced Yki pS168 change might be indirect and through upstream components of Hippo signaling.

To verify our hypothesis, we checked whether Ack interacts with other components of Hippo signaling and analyzed whether Ack affects their activity in S2 cells. By co-IP experiments, we confirmed that Ack has no interaction with Hpo or Wts. We further tested upstream components such as Mer and Ex. We found that Ack and Ex interacted with each other in both directions (Figure 3b, lane 3 and 8). In addition, overexpressed Ex can form protein complex with endogenous Ack (Supplementary Figure S2A). As Yki interacted with both Ex and Ack, we asked whether Yki mediated Ack–Ex interaction. By co-IP experiment, we found that knockdown of Yki expression by double-strand RNA (dsRNA) treatment did not affect Ack–Ex interaction and Ack-induced Ex phosphorylation (Supplementary Figure S2B). Furthermore, overexpression of Ex induced Yki S168 phosphorylation significantly, while such increase was partially suppressed by Ack in a kinase-dependent manner (Figure 3c). In addition, we did not observe any association between Ack and Mer in S2 cells (Figure 3d), supporting that Ack modulates Yki’s activity by regulating Ex function.

To explore how Ack regulates Ex function, we tested whether Ack modulates Ex phosphorylation status as its function on promoting Yki activity was kinase activity dependent. A phosphor-tyrosine (p-Y) antibody was used to detect Ex phosphorylation status when Ack was coexpressed. We confirmed the specificity of the antibody by checking the autophosphorylation status of Ack (unpublished observations). As shown in Figure 3b, Ack induced tyrosine phosphorylation of Ex specifically (compare lane 1 and 4 with lane 3). Notably, Ack did not affect Yki tyrosine phosphorylation status (Figure 3b, lane 5), further suggesting that regulation of Yki by Ack is indirect.

### Ack phosphorylates Ex on multiple tyrosine residues

**Figure 4:**
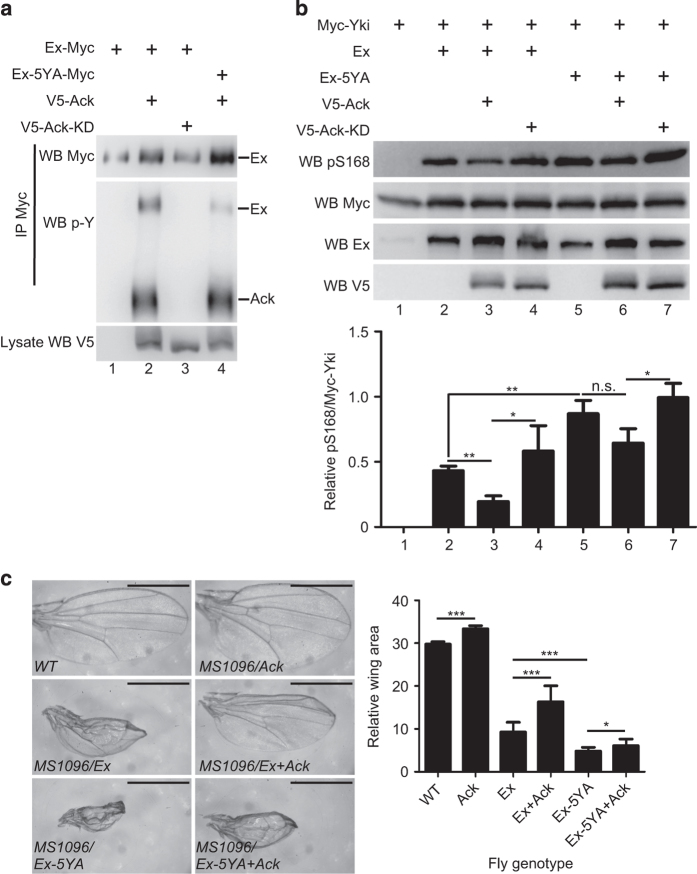
Ack suppresses Ex activity via tyrosine phosphorylation. (**a**) Ex-5YA mutation blocks majority of the phosphorylation signal induced by Ack. S2 cells expressing the indicated constructs were immunoprecipitated and probed with the indicated antibodies. Experiment was repeated three times and representative blots are shown. (**b**) 5YA mutation partially blocks Ack-induced suppression of Ex activity. S2 cells expressing the indicated constructs were probed with the indicated antibody. Yki total protein level was used as a control to examine the change of Yki phosphorylation level. Quantification of relative Yki S168 phosphorylation level is expressed as mean±s.d. (*N*=3) **P<0.01. *P<0.05. n.s., not significant. (**c**) Ex-5YA induces stronger growth defects than wild-type Ex and does not response to Ack overexpression. *Drosophila* female adult wings expressing the indicated constructs were shown. Quantification of wing area of each group is expressed as mean±s.d. (*N*=10). ***P<0.001. *P<0.05. Scale bar is 500 μm.

To identify Ex tyrosine phosphorylation sites induced by Ack, we overexpressed Myc-tagged Ex with or without Ack in S2 cells. Ex-Myc protein was then immunoprecipitated and prepared for MS analysis. Five potential tyrosine phosphorylation sites of Ex were identified, including Y227, Y423, Y679, Y766 and Y1103 (Supplementary Figure S3). To verify whether these sites are actual Ex tyrosine phosphorylation sites by Ack, we first generated an Ex mutant that contains tyrosine to alanine substitution of all potential tyrosine phosphorylation sites (Ex-5YA) and checked Ex tyrosine phosphorylation status using the p-Y antibody. We found that Ex-5YA had a much lower tyrosine phosphorylation level than wild-type Ex when cotransfected with Ack (Figure 4a). As Ex-5YA did not lose tyrosine phosphorylation signal completely, and the decrement of p-Y signal is not because of the weaker interaction between Ack and Ex-5YA, we speculate that additional tyrosine phosphorylation site(s) might exist. To identify whether these five potential tyrosine phosphorylation sites are important for Ex’s function, we coexpressed Yki with Ex or Ex-5YA in S2 cells to detect Yki pS168 change. As predicted, Ex-5YA exhibited a stronger effect on Yki activity as it almost abolished Ack-induced Ex inhibition. Ex-5YA also induced a higher Yki pS168 level than wild-type Ex (Figure 4b, compare lane 5 with lane 2). However, we noticed that Ex-5YA activity was still responsive to Ack coexpression, which may be due to the fact that the other unidentified tyrosine phosphorylation sites exist for Ack.

To verify these findings *in vivo*, we generated Ex and Ex-5YA transgenic flies using the *phiC31* integration system to ensure that all of the transgenes were expressed from the same chromosomal location (*attp* at 25C6) so that position effects on gene expression were eliminated [45]. Expression of Ex-5YA by *MS1096-Gal4* on wing discs showed stronger activity compared with the wild-type Ex in regards to the reduction in wing size (Figure 4c). Whereas Ack coexpression partially suppressed Ex-induced inhibition of wing growth, it barely had any influence on wing growth defect induced by Ex-5YA expression (Figure 4c). We believe that this phenomenon is a result from disruption of Ack regulation caused by tyrosine to alanine mutations of Ex. These findings are consistent with our observations in cultured cells and show that Ack modulates the tyrosine phosphorylation status of Ex to regulate both Yki’s activity and tissue growth.

### Y679 and Y766 are *bona fide* phosphorylation sites of Ack-induced phosphorylation

**Figure 5:**
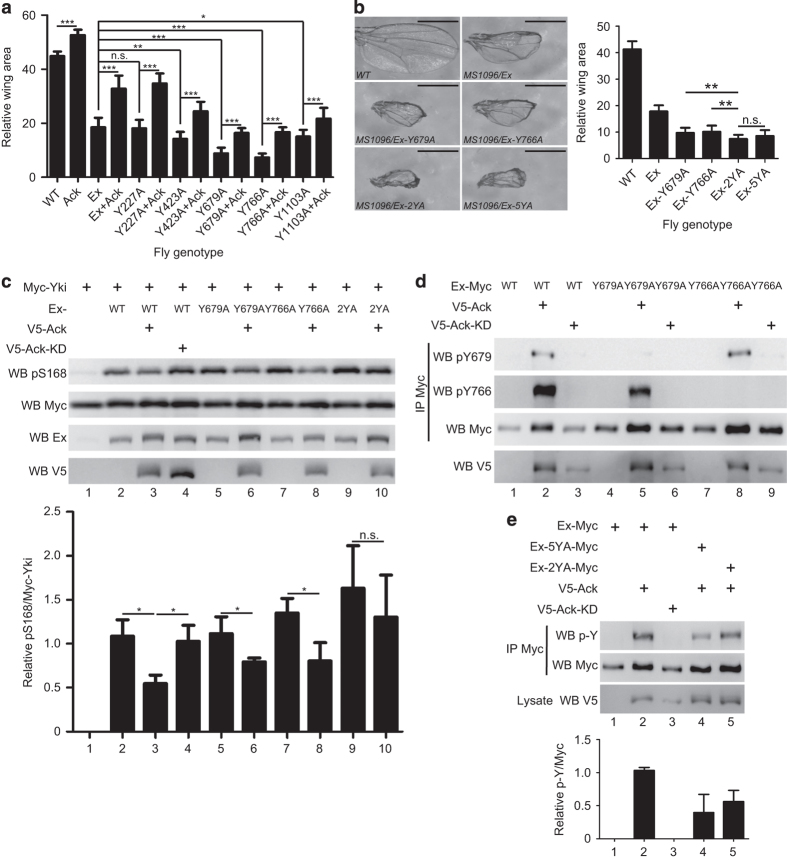
Ack phosphorylates Ex on multiple tyrosine sites to suppress Ex activity. (**a**) Ack suppressed the activity of wild-type Ex as well as Ex single site mutants. Quantification of wing area of each group is expressed as mean±s.d. (*N*=10). ***P<0.001. **P<0.01. *P<0.05. n.s., not significant. (**b**) Mutations of potential Ack-induced tyrosine phosphorylation sites affect Ex activity on inhibiting wing growth. Quantification of wing area of each group is expressed as mean±s.d. (*N*=10). **P<0.01. n.s. no significant. Scale bar is 500 μm. (**c**) S2 cells expressing the indicated constructs were probed with the indicated antibody. Yki total protein level was used as a control to examine the change of Yki phosphorylation level. Quantification of relative Yki S168 phosphorylation level is expressed as mean±s.d. (N=3). *P<0.05. n.s., not significant. (**d**) Ack induces Ex phosphorylation on Y679 and Y766. S2 cells expressing the indicated constructs were immunoprecipitated with Myc antibody and probed with the indicated antibody. (**e**) Ack-induced tyrosine phosphorylation level of Ex-2YA was lower than Ex wild type but higher than Ex-5YA. S2 cells expressing the indicated constructs were immunoprecipitated and probed with the indicated antibodies. Ex total protein level was used as a control to examine the change of Ex phosphorylation level. Quantification of relative p-Y/Myc is expressed as mean±s.d. (*N*=3).

To further explore whether a change of any of these phosphorylation sites has a critical role for Ex during growth, we generated transgenic flies expressing Ex mutants with individual tyrosine to alanine substitution. We found that flies carrying several Ex single tyrosine mutation (Y423, Y679, Y766 and Y1103) exhibited stronger growth inhibition effect than those carrying wild-type Ex. We reasoned that, by abolishing Ack-induced Ex phosphorylation, Ex’s function on growth inhibition is enhanced. However, all of the Ex mutants bearing single mutation were partially suppressed by Ack overexpression in varying degrees (Figure 5a). Notably, Ex mutants, Y679A and Y766A, exhibited stronger repressive activity on wing growth compared with the wild-type Ex and other single mutations (Figure 5a). Thus, we predicted that Y679 and Y766 double mutants would have stronger effects on Ex’s activity. To test this, we generated Ex *attp* flies containing a combination of Y679A and Y766A mutations (Ex-2YA). We found that Ex-2YA exhibited more severe growth defects than any of single mutants and in a level similar to Ex-5YA (Figure 5b). Consistently, in S2 cells, Ex-2YA overexpression-induced increase of Yki S168 phosphorylation was barely affected by Ack coexpression, whereas Ex-Y679A- and Ex-Y766A-induced Yki S168 phosphorylation is still responsive to Ack coexpression (Figure 5c). These results suggest that Y679 and Y766 sites have major roles in the regulation of Ack-mediated Ex’s function. To confirm Y679 and Y766 are *bona fide* phosphorylation sites of Ack-induced phosphorylation, we generated phospho-specific antibodies against Y679 and Y766. We found that these antibodies specifically recognize phosphorylated wild-type Ex by Ack coexpression in S2 cells, and the signal was abolished by coexpression of Ack-KD or corresponding Ex-Y679A and Ex-Y766A mutations (Figure 5d). Of note, although Y679 and Y766 are the critical sites that respond to Ack-induced regulation, the tyrosine phosphorylation level of Ex-2YA was still higher than Ex-5YA (Figure 5e), suggesting that the rest three sites are still phosphorylated by Ack. Taken these results together, we conclude that Ack suppresses Ex, regarding its functions on growth restriction, through regulating multiple sites of phosphorylation, especially Ex Y679 and Y766 residues.

### Genetic interaction between Ack and Ex/Mer

**Figure 6:**
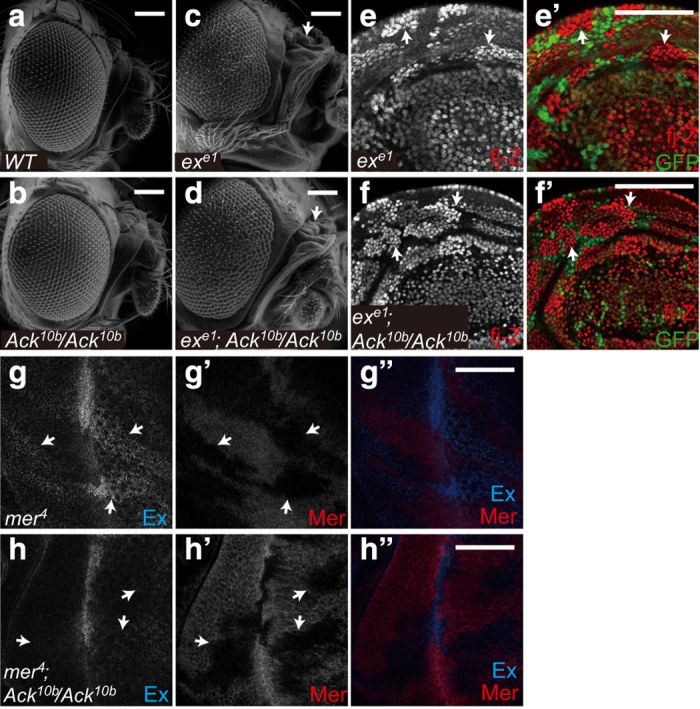
Genetic interaction between Ack and Ex/Mer. (**a**–**d**) Homozygous loss of *ack* cannot suppress *ex* mutant-induced tissue overgrowth. Scan electronic micrographs of adult fly head of the indicated genotype. Arrows indicated overgrowth in *ex* mutant. The genotypes were the following: *eyflp; ex*^*e1*^ *FRT40A/ubi-GFP FRT40A* (**c**), *eyflp; ex*^*e1*^ *FRT40A/ubi-GFP FRT40A; Ack*^*10b*^*/Ack*^*10b*^ (**d**). Scale bar is 100 μm. (**e**–**f′**) Wing discs containing *ex*^*e1*^ clones or *ex*^*e1*^*;Ack*^*10b/10b*^ clones were immunostained to show *fj-lacZ* (*fj-Z*) and GFP expression. *ex*^*e1*^ clones were marked by the loss of GFP signal. Arrows indicated upregulation of *fj-Z* expression in *ex*^*e1*^ clones. Note that homozygous loss of *ack* cannot suppress *ex* mutant-induced upregulation of *fj-Z*. The genotypes were the following: *hsflp; ex*^*e1*^ *fj-Z FRT40A/ubi-GFP FRT40A* (**e**–**e′**), *hsflp; ex*^*e1*^ *fj-Z FRT40A/ubi-GFP FRT40A; Ack*^*10b*^*/Ack*^*10b*^ (**f**–**f’**). Scale bar is 50 μm. (**g**–**h′′**) Eye discs containing *mer*^*4*^ clones or *mer*^*4*^*; Ack*^*10b/10b*^ clones were immunostained to show Ex and Mer expression. Note that homozygous loss of *ack* suppresses *mer* mutant-induced upregulation of Ex. Arrows indicated the clone boundary. The genotypes were the following: *eyflp, mer*^*4*^ *FRT19A/FRT19A* (**g**–**g′′**), *eyflp, mer*^*4*^ *FRT19A/FRT19A;;Ack*^*10b*^*/Ack*^*10b*^ (**h**–**h′′**). Scale bar is 50 μm.

To verify the genetic epistatic relationship of *ack* and *ex in vivo*, we used *ack* mutant flies generated by excision of a P-element KG00869 [46]. Surprisingly, although two Ack RNAi lines targeting different sites strongly suppress tissue growth (unpublished observations), homozygous *ack* mutant flies are viable without obvious growth defect. In addition, expression of Hippo pathway target genes did not change in *ack* null imaginal discs. Such a striking discrepancy implies that there may be potential problems for the RNAi lines. The viable property of *ack* mutant flies made it a suitable tool for examining epistatic relationships with other components in double mutant analysis. In our analysis, *ex* mutant mosaic induced massive overgrowth not only in eyes but also at the base of antenna (Figure 6, compare 6c with 6a), whereas *mer* mutant caused mild phenotype. Double mutant of *ack* and *ex* changed neither growth phenotype (Figure 6, compare 6d with 6b and c) nor *fj-lacZ* elevation in both wing and eye discs (Figure 6e–f′ and Supplementary Figure S4A–B′′) induced by loss of *ex *[47]. On the contrary, *mer* mutant-induced Ex upregulation [42, 48] was suppressed by *ack* mutant (Figure 6g–h′′). We also found that in S2 cells knockdown of Ack expression by dsRNA treatment promoted Mer expression-induced Yki pS168 level (Supplementary Figure S4C). It is interesting that loss of *ack* suppresses *mer* but not *ex* phenotype. Consistently, several previous reports also pointed out there are functional differences between Ex and Mer in Hippo signaling [49–51].

### Ack may disrupt Ex–Yki binding to promote Yki activity

We have studied the regulation of Ex by Ack extensively but the significance of Ack–Yki interaction is not shown. It has been reported that Ex interacts with Yki directly through WW–PPxY association to inhibit Yki activity [32, 33], which bypasses the Hippo pathway core kinase cascade (i.e., a phosphorylation-independent Yki regulation). We then asked whether Ack-induced tissue growth is partially resulted from a modulation of Ex–Yki association. We found that the interaction between Ex and Yki was weakened by Ack partially in a kinase activity-dependent manner (Supplementary Figure S5A, compare lane 3 and 4 with lane 2). To verify this regulation *in vivo*, we expressed Ack and Ack-KD in *hpo* clones generated by Mosaic Analysis with A Repressible Cell Marker [52, 53] system. It blocks the regulation path via the Hippo pathway core kinase cascade. As reported, induction of *hpo* mutant clones leads to an overgrowth phenotype (Supplementary Figure S5B). Overexpression of Ack (but not Ack-KD) in *hpo* clones further promotes the overgrowth phenotype compared with *hpo* clones alone (Supplementary Figure S5, compare S5C and D with S5B). Yki-S168A mutation also blocked the kinase-dependent function of the Hippo pathway. We found that Ack (but not Ack-KD) further promoted the overgrowth phenotype induced by Yki-S168A (Supplementary Figure S5E–G). *In toto*, we speculated that Ack may disrupt Ex–Yki association, thus weaken Ex-induced retention of Yki in cytosol to modulate growth as well.

**Figure 7:**
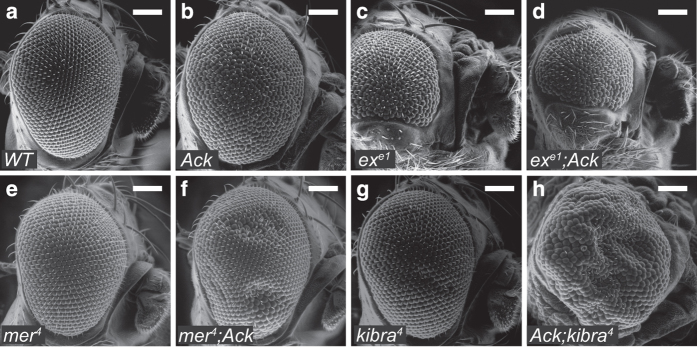
Ack promotes tissue growth mainly through regulating Ex. (**a**–**h**) Scan electronic micrograph of the adult eye of the indicated genotype was shown. Note that Ack overexpression in both *mer* (**e** and **f**) and *kibra* (**g** and **h**) mutant eye induces further growth effect but in *ex* (**c** and **d**) mutant eye did not cause dramatic change. The genotypes were the following: *eyflp, ubi-Gal4, UAS-GFP; ex*^*e1*^ *FRT40A/Gal80 FRT40A* (**c**), *eyflp, ubi-Gal4, UAS-GFP; ex*^*e1*^ *FRT40A/Gal80 FRT40A; UAS-V5-Ack* (**d**), *eyflp, mer*^*4*^ *FRT19A/Gal80 FRT19A; ubi-Gal4, UAS-GFP* (**e**), *eyflp, mer*^*4*^ *FRT19A/Gal80 FRT19A; ubi-Gal4, UAS-GFP; UAS-V5-Ack* (**f**), *eyflp, ubi-Gal4, UAS-GFP; FRT82B kibra*^*4*^*/FRT82B Gal80* (**g**) and *eyflp, ubi-Gal4, UAS-GFP; UAS-V5-Ack; FRT82B kibra*^*4*^*/FRT82B Gal80* (**h**). Scale bar is 100 μm.

Taken together, our data lead to a model in which Ack promotes growth via suppressing Ex. To further determine whether this is true *in vivo*, we overexpressed Ack in *ex*, *mer* or *kibra* mutants using the Mosaic Analysis with A Repressible Cell Marker system. In control group, flies overexpressing Ack promoted adult eye overgrowth compared with wild-type flies (Figure 7a and b). Ack overexpression in *ex* mutant clones failed to cause dramatic change (Figure 7c and d), while Ack further promoted growth in the absence of *mer* or *kibra* (Figure 7e–h). These results suggest that Ack modulates growth mainly through negatively regulating Ex.

## Discussion

In this study, we report the identification of non-receptor tyrosine kinase Ack as a novel regulator of the Hippo pathway. Using Yki WW domain as a bait, we found that Ack interacts with Yki from our MS analysis, consistent with results from two earlier studies [10, 38]. We confirmed the interaction between Ack and Yki, and found that Ack modulates Yki activity (Figures 1 and 2) in a kinase activity-dependent manner to promote tissue growth.

Schoenherr *et al.* [10] reported an anti-apoptotic function of Ack, which is regulated by Yki. They proposed that Ack cannot promote Yki activity and nuclear localization, as they found that overexpression of Ack by *GMR-Gal4* failed to promote Ex-Z expression. Consistently, we also found no change of Ex-Z in this condition. However, we noticed that the protein levels of other two Yki targets, Diap1 and cyclin E, were increased in Ack-overexpressing eye discs (unpublished observations). Taken together with the adult phenotype, we concluded that Ack modulates Yki activity in eye discs. The anti-apoptotic function of Ack may be partially explained by increased Yki activity since increased Diap1 protein level may inhibit cell death [54, 55]. The reason why Ex-Z shows no change may be due to varied sensitivities of the reporter in different tissues.

Our results imply that Yki might not be a substrate for Ack tyrosine phosphorylation (Figure 3b, lane 5). To elucidate the mechanism of how Ack promotes growth, we examined Hippo pathway upstream components and found that Ack interacts with Ex and induces Ex tyrosine phosphorylation, indicating that Ack might function through Ex. We further confirmed that Ack mediates growth via Ex phosphorylation on Tyr679 and Tyr766 (Figures 4 and 5). Interestingly, Ex overexpression induces Yki Ser168 phosphorylation dramatically. We suppose Ex phosphorylation induced by Ack reduces its ability to efficiently promote the Hpo/Wts core kinase cascade, thereby Yki is less phosphorylated. However, Yki may not be the only target that mediates Ack activity during tissue growth. Ack may have ex-dependent but Hippo pathway-independent function. One reason is that coexpression of Ack with YAP-S94A/S127A mutation form still could promote cell proliferation [10]. Another one is there is Hippo pathway-independent function of Ex, such as regulating receptor endocytosis [56]. Based upon our findings, it is intriguing to speculate that Ack regulates Hippo signaling in a similar way in mammals. Like most of other Hippo pathway components, Ack is highly conserved from *Drosophila* to human [1]. So far, no comparable Ex homologs have been found in mammals. Willin/FRMD6 was considered as the human Ex ortholog and its expression has been shown to activate Hippo signaling [57], while it lacks the C-terminus, where the most important phosphorylated tyrosine residues Y679 and Y766 are located. Therefore, it will be interesting to determine whether Ack inhibits Willin/FRMD6 in mammals.

It was previously reported that ACK1 transduces extracellular signals to cytosolic and nuclear effectors to promote cell survival and growth, and several somatic ACK1 mutations discovered in tumors could increase the ability of tumor cells to proliferate and migrate [58]. Whether Hippo signaling is affected in these tumor cells is an interesting question to explore. Recently, several small molecules that act as potential Ack inhibitors have been reported [59–63], and they may lead to effective drugs targeting Ack in the future [64, 65]. Our study thus provides the basis of the mechanism underlying these therapeutic approaches for curbing tumorigenesis.

## Materials and Methods

### Clones, mutants, *Drosophila* stocks and genetics

The full-length Ack DNA fragment (3 222 bp) was amplified from a BDGP DGC clone (clone ID: GH10777) and was inserted into *pUAST-V5* or *pUAST-Flag* vector. Ack and Ex mutants that contain point mutation were generated by PCR-based site-directed mutagenesis.

Ex transgenic flies were generated by site-specific integration into the fly genome at 25C6 attp locus. *ack* null allele (*Ack*^*10b*^) was a kind gift from Nicholas Harden, Simon Fraser University. *kibra* null allele (*kibra*^*4*^) was a kind gift from Hugo Stocker, Institute of Molecular Systems Biology. *fj-lacZ* was a kind gift from Kenneth D. Irvine, The State University of New Jersey. Other RNAi or null alleles used in this study were as following: *UAS-AckRNAi* (NIG 14992R1 and VDRC 39857), *UAS-YkiRNAi* (VDRC 40497), *ex*^*e1*^ [66] and *mer*^*4*^ [67]. *MS1096-Gal4*, *GMR-Gal4*, *hh-Gal4*, *hpo*^*BF33*^, *Ex-lacZ*, *diap1-GFP4.3* and *bantam sensor mic32-GFP* were described previously [40, 68]. Flies and crosses were cultured at 25 °C unless otherwise indicated.

### Protein pull-down and MS screen

For pull-down experiment, *Drosophila* S2 cells were cultured for 3 days (10 dishes) and then collected and lysed in immunoprecipitation (IP) buffer (50 mm Tris-HCl, pH 8.0, 100 mm NaCl, 1% NP-40, 10% Glycerol, 1.5 mm EDTA, 10 mm NaF, 1 mm Na_3_VO_4_) with protease inhibitor cocktail (Sigma, St Louis, MO, USA). Cell lysates were pre-cleared using Glutathione sepharose (GE Healthcare, Little chalfont, UK) and then incubated with 200 μg immobilized GST-WW protein, which was expressed in BL21 *E. coli* and was purified with Glutathione Sepharose. GST protein was used as a negative control. Proteins in pull-down samples were separated by SDS-PAGE, and were stained using Colloidal Blue staining (Invitrogen, Carlsbad, CA, USA). Compared with control sample, specific bands were selected for liquid chromatography coupled with tandem MS analysis in Protein Centre, SIBCB.

### Cell culture, transfection and co-IP

S2 cells were cultured in *Drosophila* Schneider’s Medium (Invitrogen) with 10% fetal bovine serum, 100 U ml^−1^ of penicillin and 100 mg ml^−1^ of Streptomycin. Plasmid transfection was carried out using LipofectAMINE (Invitrogen) according to the manufacturer’s instructions. The constructs transfected in S2 cells were all pUAST expression vectors unless otherwise indicated. A construct of *ubiquitin-Gal4* was cotransfected with *pUAST* expression vectors for all transfection experiments.

For co-IP experiment, S2 cells expressing the indicated constructs were collected and lysed in IP buffer (50 mm Tris-HCl, pH 8.0, 100 mm NaCl, 1% NP-40, 10% Glycerol, 1.5 mm EDTA, 10 mm NaF, 1 mm Na_3_VO_4_) with protease inhibitor cocktail (Sigma). Cell lysates were then divided into two parts, and 2 μg antibody was used to do co-IP in each part. Proteins in IP samples were then washed with IP buffer and subjected for western blot analysis.

### Western blot, immunostaining and luciferase reporter assay

Western blot analysis and immunostaining were performed as previously described [40]. Primary antibodies used in western blot and immunostaining were as follows: mouse anti-Myc (1:5 000, Sigma), mouse anti-Flag (1:5 000, Sigma), mouse anti-V5 (1:5 000, Invitrogen), mouse anti-HA (1:5 000, Sigma), mouse anti-phospho tyrosine (1:1  000, Cell Signaling Technology, Danvers, MA, USA), rabbit anti-Ack (1:500), rat anti-Ci (1:500, Developmental Studies Hybridoma Bank, DSHB), rabbit anti-Ex (a gift from Allen Lauhgon, University of Wisconsin), guinea pig anti-Mer (a gift from Richard G. Fehon, University of Chicago) and rabbit anti-lacZ (1:1 000, Invitrogen). Rabbit anti-Ack (556-1073AA) was generated by Abclonal (Wuhan, China). Rabbit anti-phospho Ex (Y679 and Y766) was generated by Abclonal, with peptide as antigen (Y679, ESEKSSHpYGMFQPQK; Y766, SLHSDCDpYVTLPLGD). Rabbit anti-phospho Yki (S168) was generated by Abgent (Suzhou, China) with antigen peptide HHSRARpSSPAC.

For Luciferase reporter assay, the *3xSd2-Luc* reporter was described previously [40]. The Dual-Glo™ luciferase assay system (Promega, Madison, WI, USA) was used according to the manufacturer's instructions.

### RNA interference in *Drosophila* S2 cells

dsRNA was designed and synthesized according to standard protocol. To perform knockdown experiment, S2 cells were diluted into 1×10^6^ cells per ml with serum-free medium for 1 h starvation with 15 μg ml^−1^ indicated dsRNA.

### Mapping Ex phosphorylation sites

To identify Ack-induced Ex phosphorylation sites, S2 cells were transfected with Ex-Myc or Ex-Myc and V5-Ack (10 dishes for each group). Cells were collected 48 h after transfection and were lysed in IP buffer. Ex-Myc protein was immunoprecipitated by 50 μg immobilized mouse anti-Myc antibody (Sigma) and subjected to MS analysis at the Protein Center, SIBCB. The candidate sites were identified by the increased phosphorylation abundance in the Ex-Myc and V5-Ack transfected cells versus Ex-Myc transfected cells.

### Statistical analysis

All of the data in this study were expressed as the mean±s.d. and were analyzed using the Student’s *t*-test. The results were considered as statistically significant if *P*<0.05.

### Microscopy and data analysis

Fluorescent microscopy was performed on a Leica LAS SP5 confocal microscope; confocal images were obtained using the Leica AF Lite system (Leica Microsystems, Wetzlar, Germany). Images were processed in Photoshop CS.
