# Supplementary material for: Ack promotes tissue growth via phosphorylation and suppression of the Hippo pathway component Expanded
Source: Cell Discov. 2016 Feb 23;2:15047–. doi: 10.1038/celldisc.2015.47 (PMC4860957; doi:10.1038/celldisc.2015.47)
Supplement: Supplementary Figure S1 [file celldisc201547-s1.pdf]

Figure S1 A table of partial MS results.

	Peptide	UniqPeptide
Warts	6	6
Hippo	2	2
Mop	4	4
WWBP	42	6
Ack	71	30
