# Supplementary material for: Ack promotes tissue growth via phosphorylation and suppression of the Hippo pathway component Expanded
Source: Cell Discov. 2016 Feb 23;2:15047–. doi: 10.1038/celldisc.2015.47 (PMC4860957; doi:10.1038/celldisc.2015.47)
Supplement: Supplementary Figure S2 [file celldisc201547-s2.pdf]

Figure S2   Ack interactes with Ex.

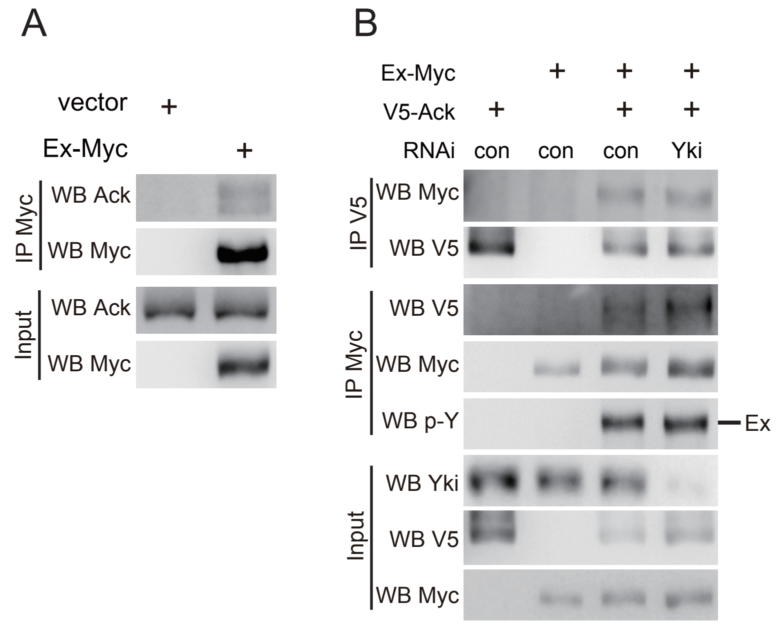

(A) Endogenous Ack interacts with overexpressed Ex-Myc. S2 cells expressing the indicated constructs were immunoprecipitated and probed with the indicated antibodies. (B) Ack-Ex interaction is not dependent on Yki. S2 cells expressing the indicated constructs were immunoprecipitated and probed with the indicated antibodies.
