# Supplementary material for: Ack promotes tissue growth via phosphorylation and suppression of the Hippo pathway component Expanded
Source: Cell Discov. 2016 Feb 23;2:15047–. doi: 10.1038/celldisc.2015.47 (PMC4860957; doi:10.1038/celldisc.2015.47)
Supplement: Supplementary Figure S3 [file celldisc201547-s3.pdf]

Figure S3 MS result of Ex phosphorylation sites.

| Gene names | Number of Phospho (STY) | Amino acid | Sequence window | Modified sequence |
|---|---|---|---|---|
| ex | 1 | S | AAAGAGSSSSSMFARSRSDDNILNSLDLLPK | _S(ph)RSDDNILNSLDLLPK_ |
| ex | 1;2 | S | KPIAVVAPKPDSPPCSPPVPPAPIPAPPPAI | _PIAVVAPKPDS(ph)PPCS(ph)PPVPPAPIPAPPPAIR_ |
| ex | 1;2 | S | KPIPKPIAVVAPKPDSPPCSPPVPPAPIPAP | _PIAVVAPKPDS(ph)PPCS(ph)PPVPPAPIPAPPPAIR_ |
| ex | 1 | S | AGAGSSSSSMFARSRSDDNILNSLDLLPKGK | _SRS(ph)DDNILNSLDLLPK_ |
| ex | 1 | Y | PSRKTVSSSLHSDCDYVTLPLGDQGEEEVDQ | _TVSSSLHSDCDY(ph)VTLPLGDQGEEEVDQPPAPPPPYSAR_ |
| ex | 1 | Y | YLAMQVSPAIYRSTPYLPLTLSTHSRYASTQ | _STPY(ph)LPLTLSTHSR_ |
| ex | 1 | Y | KREEEEAESNRLHASYACSRSLLLPYKSKNE;REEEEAAESQRLHASYACSRSLLLPYKSKNE | _LHASY(ph)ACSR_ |
| ex | 1 | Y | TGQSSEIAESEKSSHYGMFQPQKLEETHVQH | _SSHY(ph)GMFQPQK_ |
| ex | 1 | S | HKREEEEAESNRLHASYACSRSLLLPYKSKN;KREEEEAAESQRLHASYACSRSLLLPYKSKN | _LHAS(ph)YACSR_ |
| ex | 1 | Y | KISKRANERMLRLSTYVASTSKRETIPLPPS;KRLSSANERMLRLSTYVASTSKREPLPLPPL | _LSTY(ph)VASTSK_ |
| ex | 2 | T | KNEQRISVISSTSSNTTSGIVSDRVHSEDEL | _ISVISSTS(ph)SNT(ph)TSGIVSDR_ |
| ex | 1 | S | DLGDAPPGTSNSKDDSGEETSASPSNGGRGL | _DDS(ph)GEETSASPSNGGR_ |
