# Supplementary material for: Ack promotes tissue growth via phosphorylation and suppression of the Hippo pathway component Expanded
Source: Cell Discov. 2016 Feb 23;2:15047–. doi: 10.1038/celldisc.2015.47 (PMC4860957; doi:10.1038/celldisc.2015.47)
Supplement: Supplementary Figure S4 [file celldisc201547-s4.pdf]

Figure S4   Genetic interaction between Ack and Ex.

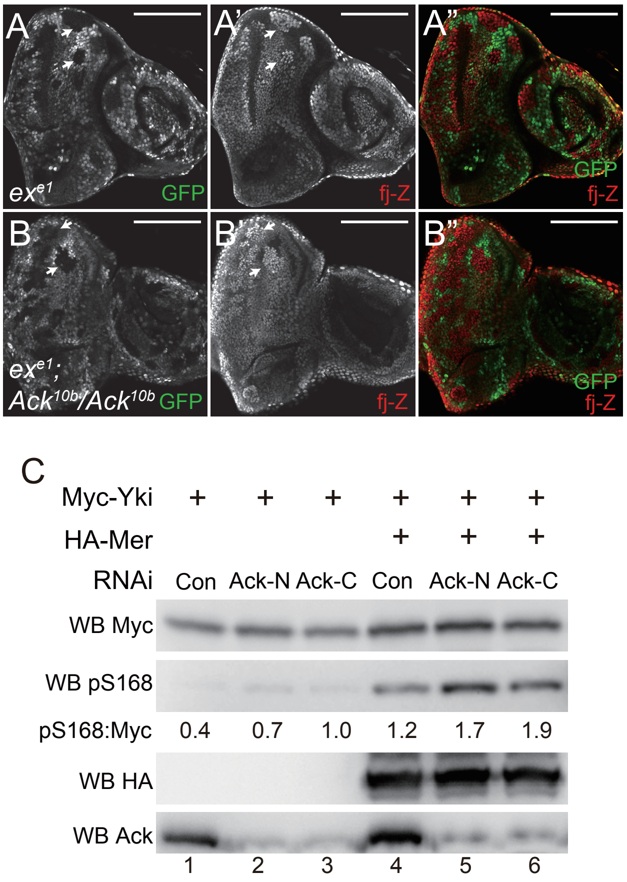

(A-B”) Eye discs containing *ex* clones or ex^{e1};$Ack^{10b/10b}$ clones were immunostained to show fj-lacZ (fj-Z) and GFP expression. ex^{e1} clones were marked by the loss of GFP signal. Arrows indicated upregulation of fj-Z expression in ex clones. Note that homozygous loss of *ack* cannot suppress ex^{e1} mutant induced upregulation of fj-Z. The genotypes were the following: *hsflp;* ex^{e1} *fj-Z FRT40A/ubi-GFP FRT40A* (A-A”), *hsflp;* ex^{e1} *fj-Z FRT40A/ubi-GFP FRT40A;* Ack^{10b}/Ack^{10b} (B-B”). Scale bar is 100 μm. (C) Knockdown of Ack expression promoted Mer induced Yki pS168 level. S2 cells expressing the indicated constructs and treated with the indicated dsRNA were probed with the indicated antibodies. Experiment was repeated three times and representative blots are shown. pS168 and Myc signal level was calculated using Image J software.
