# Supplementary material for: Ack promotes tissue growth via phosphorylation and suppression of the Hippo pathway component Expanded
Source: Cell Discov. 2016 Feb 23;2:15047–. doi: 10.1038/celldisc.2015.47 (PMC4860957; doi:10.1038/celldisc.2015.47)
Supplement: Supplementary Figure S5 [file celldisc201547-s5.pdf]

Figure S5 Ack may disrupt Ex-Yki association to promote Yki activity.

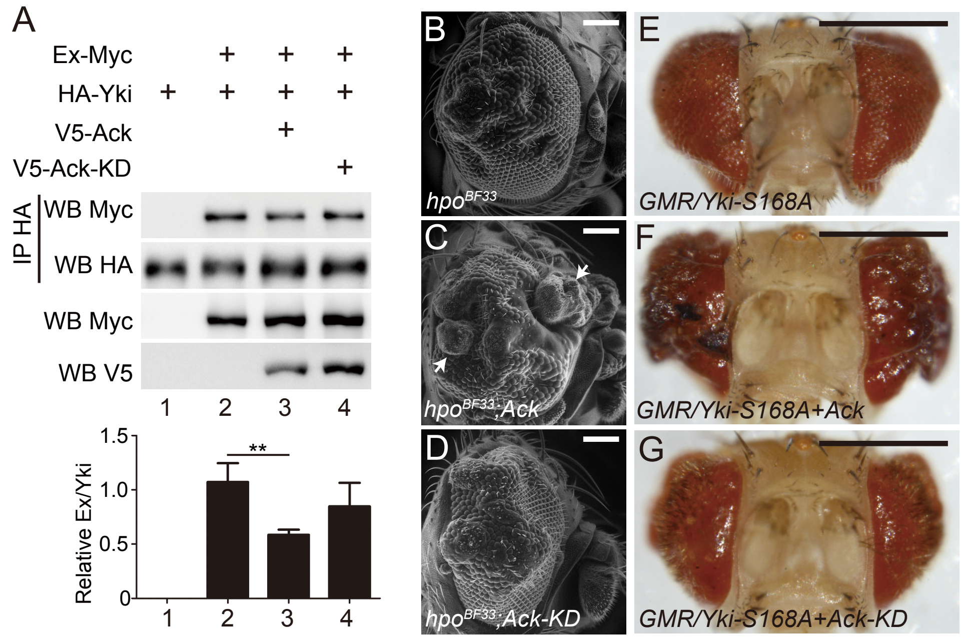

(A) Ack disrupted Ex-Yki association. S2 cells expressing the indicated constructs were immunoprecipitated and probed with the indicated antibodies. Quantification of relative protein level Ex/Yki is expressed as mean±S.D. (N=3) ** p<0.01. (B-D) Scan electronic micrographs of the adult eyes of indicated genotypes. Arrows indicated Ack overexpression in *hpo* mutant clones induces further growth phenotype. The genotypes were the following: *eyflp, ubi-Gal4, UAS-GFP; FRT42D hpo^{BF33}/FRT42D Gal80* (B), *eyflp, ubi-Gal4, UAS-GFP; FRT42D hpo^{BF33}/FRT42D Gal80; UAS-V5-Ack* (C), *eyflp, ubi-Gal4, UAS-GFP; FRT42D hpo^{BF33}/FRT42D Gal80; UAS-V5-Ack-KD* (D). Scale bar is 100 μm. (E-G) Overexpression of Ack promoted Yki-S168A induced overgrowth phenotype. Experiments was repeated and representative pictures were shown. Scale bar is 500μm.
